# Deep learning supports the differentiation of alcoholic and other-than-alcoholic cirrhosis based on MRI

**DOI:** 10.1038/s41598-022-12410-2

**Published:** 2022-05-18

**Authors:** Julian A. Luetkens, Sebastian Nowak, Narine Mesropyan, Wolfgang Block, Michael Praktiknjo, Johannes Chang, Christian Bauckhage, Rafet Sifa, Alois Martin Sprinkart, Anton Faron, Ulrike Attenberger

**Affiliations:** 1 Department of Diagnostic and Interventional Radiology, Quantitative Imaging Lab Bonn (QILaB), University Hospital Bonn, Venusberg-Campus 1, 53127 Bonn, Germany; 2 Department of Radiotherapy and Radiation Oncology, University Hospital Bonn, Venusberg-Campus 1, 53127 Bonn, Germany; 3 Department of Neuroradiology, University Hospital Bonn, Venusberg-Campus 1, 53127 Bonn, Germany; 4 Department of Internal Medicine I, Center for Cirrhosis and Portal Hypertension Bonn (CCB), University Hospital Bonn, Venusberg-Campus 1, 53127 Bonn, Germany; 5 Institute for Computer Science, University of Bonn, Endenicher Allee 19C, 53113 Bonn, Germany; 6 Media Engineering Department, Fraunhofer IAIS, Schloss Birlinghoven 1, 53757 Sankt Augustin, Germany

**Keywords:** Liver cirrhosis, Gastroenterology, Hepatology, Computational science, Machine learning

## Abstract

Although CT and MRI are standard procedures in cirrhosis diagnosis, differentiation of etiology based on imaging is not established. This proof-of-concept study explores the potential of deep learning (DL) to support imaging-based differentiation of the etiology of liver cirrhosis. This retrospective, monocentric study included 465 patients with confirmed diagnosis of (a) alcoholic (n = 221) and (b) other-than-alcoholic (n = 244) cirrhosis. Standard T2-weighted single-slice images at the caudate lobe level were randomly split for training with fivefold cross-validation (85%) and testing (15%), balanced for (a) and (b). After automated upstream liver segmentation, two different ImageNet pre-trained convolutional neural network (CNN) architectures (ResNet50, DenseNet121) were evaluated for classification of alcohol-related versus non-alcohol-related cirrhosis. The highest classification performance on test data was observed for ResNet50 with unfrozen pre-trained parameters, yielding an area under the receiver operating characteristic curve of 0.82 (95% confidence interval (CI) 0.71–0.91) and an accuracy of 0.75 (95% CI 0.64–0.85). An ensemble of both models did not lead to significant improvement in classification performance. This proof-of-principle study shows that deep-learning classifiers have the potential to aid in discriminating liver cirrhosis etiology based on standard MRI.

## Introduction

As the end stage of chronic liver disease, liver cirrhosis is a major health issue. In particular, patients with liver cirrhosis have a concomitant risk for the development of hepatocellular carcinoma as well as complications arising from decompensation such as variceal bleeding or hepatic encephalopathy. Overall, the prevalence of chronic liver disease is internationally expected to grow within the next decades^1–3^.

Many factors that contribute to the development of cirrhosis have been identified. The most common etiologies are alcohol consumption, obesity, and chronic viral infections^3^. Thereby, identification of the underlying cause of disease is crucial as appropriate treatment may not only stop disease from progression, but in certain cases also may facilitate regression of fibrosis^2,4,5^. In the majority of countries, alcohol consumption still represents the leading cause of liver disease and is directly related to liver mortality^3,6^. In these patients, alcohol abstinence was shown to be critical for long-term outcome, may improve various aspects of disease severity and is also fundamental with regard to potential liver transplantation^4,7,8^. However, until experiencing severe complications such as acute decompensation, many patients with cirrhosis are unaware of their underlying condition^2,3^. Liver cirrhosis results from chronic inflammation and hence leads to distinct changes in hepatic morphology, which in part can be detected by high-resolution imaging methods such as MRI^9,10^.

Although it has been described that the micro- and macroscopic appearance of cirrhosis in medical imaging varies to some extent according to the underlying etiology, the use of imaging features as a means to determine the cause of the disease has not been established yet^9,11,12^. In a previous study, a convolutional neural network (CNN) was already shown to be able to detect liver cirrhosis based on standard clinical MRI sequences with expert-level accuracy irrespective of etiology^13^. Therefore, the aim of this proof-of-concept study was to investigate deep learning for standard MRI based characterization of disease etiology, differentiating alcoholic- versus other-than-alcoholic cirrhosis.

## Materials and methods

### Dataset

**Figure 1:**
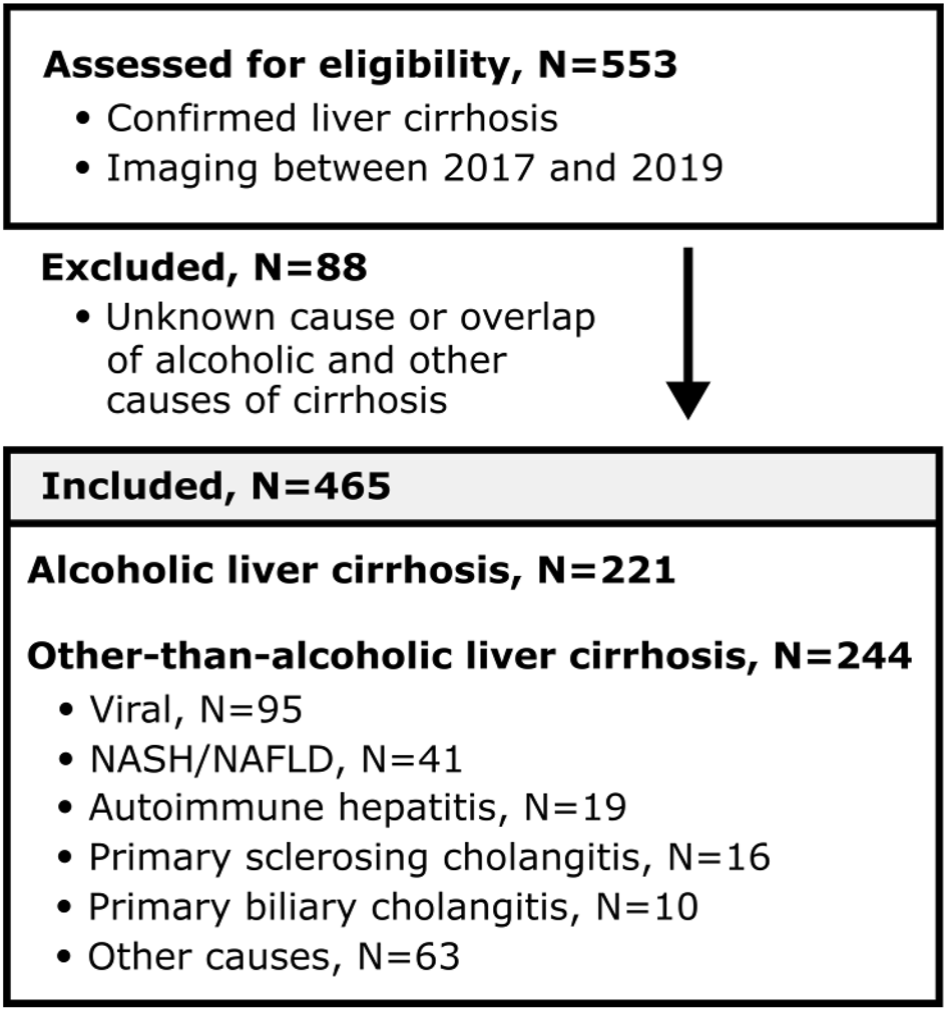
Study inclusion flow chart. Patients with confirmed diagnosis of liver cirrhosis who underwent liver MRI between 2017 and 2019 were evaluated for inclusion. Patients with unknown causes of liver cirrhosis and with documented overlap of alcoholic liver cirrhosis with other causes were excluded from the analysis. The final cohort consisted of 465 patients. Those patients were separated according to liver cirrhosis etiology into patients with (**a**) alcoholic liver cirrhosis (N = 221) and (**b**) other-than-alcoholic liver cirrhosis (N = 244). Abbreviations: *NAFLD/NASH* non-alcoholic fatty liver disease/non-alcoholic steatohepatitis.

The study was approved by the Ethics Committee at the Medical Faculty of the Rheinische Friedrich-Wilhelms-Universität Bonn and the need for written informed consent was waived due to its retrospective, single-center nature. The research was performed in accordance with the Declaration of Helsinki. Patients with confirmed diagnosis of liver cirrhosis, defined by clinical manifestations of liver cirrhosis (e.g. presence of dermal features, ascites, splenomegaly or hyperestrogenism), laboratory parameters (e.g. presence of parameters of hepatocyte damage or impaired hepatic synthesis), and/or histopathological criteria, who underwent liver MRI for diagnostic purposes between 2017 and 2019 at the Department of Diagnostic and Interventional Radiology at the University Hospital of Bonn, were evaluated for inclusion. The clinical information management system of the relevant institution was used to derive clinical characteristics of the study population including the respective cause of liver cirrhosis. Patients with unknown causes of liver cirrhosis and with overlap of alcoholic and other causes were excluded. The final cohort was separated according to the underlying cause of liver cirrhosis into (a) patients with alcoholic liver cirrhosis and (b) other-than-alcoholic liver cirrhosis (Fig. 1).

### Image segmentation and classification

All patients underwent a standardized imaging protocol including a standard clinical respiratory triggered multi-slice turbo spin echo sequence with non-cartesian k-space filling (T2 MultiVane XD) on a clinical 1.5 Tesla (Ingenia 1.5 T, Philips Healthcare, Best, the Netherlands) or 3.0 Tesla (Ingenia 3.0 T, Philips Healthcare, Best, the Netherlands) scanner. This sequence was shown to be suitable for deep learning-based detection of liver cirrhosis in a previous study^13^. Similar to the proposed approach for cirrhosis detection, a single cross-sectional image at the level of caudate lobe was exported, followed by liver segmentation performed by a U-net style convolutional neural network (CNN) with ResNet34 as backbone that was developed and validated on a dataset of 713 single slice T2- weighted MRI images^13^. The images were first normalized and image augmentation was applied during training. Supplementary information on imaging parameters and image preprocessing can be found in Supplement S1 and S2.

For imaging development of a classification CNN that differentiates patients with alcoholic liver cirrhosis and other-than-alcoholic liver cirrhosis, data were randomly split into a training (85%) and a hold-out test set (15%). Training was performed with fivefold cross-validation. An ensemble of the cross-validated models was applied to the test set.

A CNN with residual connections (ResNet50) with ImageNet pre-trained parameters was used, as this established architecture was shown to be suitable for the detection of liver cirrhosis^13,14^. To investigate whether the use of a different pre-trained architecture than ResNet50 or an ensemble of two architectures is beneficial, a CNN with dense connections (DenseNet121) was additionally evaluated, which has fewer trainable parameters and is less computationally intensive compared to ResNet50^15^.

Furthermore, two different training strategies were evaluated for ResNet50 and DenseNet121 in order to examine whether altering the pre-trained parameters of the CNN may impact classification performance. First, both networks were trained with frozen pre-trained parameters of the convolutional layers. In a second subsequent training run, the pre-trained convolutional layers of both networks were unfrozen with descending learning rates from the last to the first layer at several stages. Training was performed with Adam optimization, a cyclical learning rate scheme, and cross-entropy loss function. Supplementary information on the experimental design and hyper-parameters used for training are provided in Supplement S3.

Image regions that were particularly relevant to the classification task were highlighted by generating gradient-weighted class activation maps (Grad-CAM) for the test set^16^.

### Statistical analysis

Prism 8 (GraphPad software), SPSS Statistics 24 (IBM), MedCalc 20.014 (MedCalc Software Ltd) and Scikit-learn 0.23.2^17^ were used for statistical analysis. Patient characteristics are expressed as frequencies or means with standard deviation, as appropriate. Classification accuracy (ACC), as well as receiver operating characteristic (ROC) analyses was performed for the cross-validation and the test sets for both studied CNN architectures (ResNet50, DenseNet121) and both training strategies (frozen, unfrozen). For the test set, 95% confidence intervals were determined for ACC and AUC values. ROC and precision-recall curves were generated^18^. Grad-CAM images were visually inspected by one experienced radiologist (A.F.) and highlighted regions were categorized according to their anatomical localization as being predominantly situated in the right liver lobe, the left liver lobe, the portal region, the caudate lobe, or in the image background. Resulting categorical data were compared using either Fisher`s exact test (for a cell count of ≤ 5) or χ^2^ test (for a cell count > 5), as appropriate. The two-sided t-Test was used to compare differences between groups regarding continuous variables. *P* < 0.05 was set as the level of statistical significance.

## Results

### Baseline characteristics of the study population

A total of 465 patients (203 female; mean age, 60 ± 11 years) were included. Of those, 47.5% (221/465) of patients had alcoholic liver cirrhosis. 52.5% (244/465) of patients had other-than-alcoholic liver cirrhosis.

Liver biopsy was performed in 64.8% (301/465) of patients. The most common causes of non-alcohol related liver cirrhosis were viral hepatitis (39%, 95/244), non-alcoholic fatty liver disease or non-alcoholic steatohepatitis (17%, 41/244), and autoimmune hepatitis (8%, 19/244). In 5% (12/244) of patients with other-than-alcoholic liver cirrhosis, etiology of liver disease was multifactorial. Causes of liver cirrhosis of the entire study population are summarized in detail by Table 1. No significant differences regarding age (61 ± 9 years vs. 59 ± 13 years, *P* = 0.110) and gender distribution (48%, 105/221 female patients vs. 40%, 98/244 female patients, *P* = 0.111) were observed between patients with alcoholic and other-than-alcoholic liver cirrhosis. There was no difference in weight between patients with alcoholic and other-than-alcoholic cirrhosis (80.1 ± 20.4 kg vs. 80.1 ± 17.7 kg, *P* = 0.972). Values for γ-glutamyltransferase were higher in patients with alcoholic cirrhosis compared to patients with other-than-alcoholic cirrhosis (208.8 ± 264.1 U/l vs. 147.4 ± 166.1 U/l, *P* = 0.003).

**Table 1:** Liver cirrhosis etiology.

Etiology of liver cirrhosis	Number of patients (%)
Alcoholic liver cirrhosis	221 (48%)
Other-than-alcoholic liver cirrhosis	244 (52%)
Hepatitis B virus	26 (6%)
Hepatitis C virus	69 (15%)
Fatty liver disease (NAFLD/NASH)	41 (9%)
Autoimmune hepatitis	19 (4%)
Primary sclerosing cholangitis	16 (3%)
Drug-induced	13 (3%)
Primary biliary cholangitis	10 (2%)
Portal vein thrombosis	9 (2%)
Nutritional	9 (2%)
Budd-Chiari syndrome	9 (2%)
Hemochromatosis	5 (1%)
Idiopathic	5 (1%)
Sinusoidal obstruction syndrome	3 (1%)
Secondary sclerosing cholangitis	3 (1%)
Alpha-1 Antitrypsin Deficiency	3 (1%)
Wilson disease	2 (< 1%)
Congestive hepatopathy	1 (< 1%)
Sarcoidosis	1 (< 1%)

Underlying causes of liver cirrhosis are reported for the entire study population (N = 465) as total numbers as well as percentages of the entire study cohort.

*NAFLD/NASH* non-alcoholic fatty liver disease/non-alcoholic steatohepatitis.

### Classification of liver cirrhosis etiology

Segmented images of the entire study population were randomly subdivided into a training (N = 396; 174 female; mean age, 60 ± 12 years), and a test set (N = 69; 29 female; mean age, 59 ± 10 years), with training sets being further split for fivefold cross-validation, balanced for patients with alcoholic and non-alcoholic cause of liver cirrhosis.

Trained with frozen parameters, a mean accuracy (ACC) and mean area under the curve (AUC) of 0.69 and 0.78 was observed for ResNet50 and a mean ACC of 0.66 and a mean AUC of 0.78 was observed for DenseNet121 for all 5 validation splits (Table 2). With unfrozen pre-trained parameters, mean ACC values of 0.74 and 0.71 and mean AUC values of 0.83 and 0.82 were obtained for ResNet50 and DenseNet121 on cross-validated training data, respectively.

**Table 2:** Classification performance of the cross-validation and testing of the CNN architectures trained with frozen and unfrozen pre-trained parameters.

	Frozen pre-trained parameters						Unfrozen pre-trained parameters					
	ResNet50		DenseNet121		Ensemble		ResNet50		DenseNet121		Ensemble	
	AUC	ACC	AUC	ACC	AUC	ACC	AUC	ACC	AUC	ACC	AUC	ACC
**Training + validation**												
Split 1	0.737	0.684	0.764	0.671	0.776	0.658	0.798	0.696	0.780	0.671	0.807	0.709
Split 2	0.774	0.658	0.751	0.646	0.768	0.747	0.773	0.722	0.839	0.684	0.798	0.684
Split 3	0.800	0.722	0.797	0.646	0.815	0.722	0.864	0.785	0.817	0.772	0.876	0.797
Split 4	0.821	0.709	0.822	0.658	0.852	0.684	0.879	0.785	0.858	0.722	0.870	0.759
Split 5	0.742	0.671	0.756	0.684	0.770	0.722	0.822	0.722	0.805	0.696	0.813	0.709
Mean	0.775	0.689	0.778	0.661	0.796	0.707	0.827	0.742	0.820	0.709	0.833	0.732
**Test**												
	0.819	0.739	0.801	0.739	0.838	0.754	0.823	0.754	0.786	0.696	0.813	0.710

Classification accuracy and AUC values for each validation split of the cross-validation and mean over all splits. The classification accuracy and AUC values of ensembles of the cross-validated models on the test set.

*AUC* area under the curve, *ACC* accuracy.

On test data, the classification performance of ResNet50 was higher than DenseNet121 when training with unfrozen parameters, however the difference was not statistically significant (Value and 95% CI: AUC_ResNet50_ 0.82 [0.71–0.91] vs. AUC_DenseNet121_ 0.79 [0.67–0.88], *P* = 0.40; ACC_ResNet50_ 0.75 [0.64–0.85] vs. ACC_DenseNet121_ 0.70 [0.57–0.80], *P* = 0.26). Also, training with unfrozen parameters did not differ significantly compared to frozen parameters for both ResNet50 and DenseNet121 (AUC_ResNet50_, 0.82 [0.71–0.91] vs. 0.82 [0.71–0.90], *P* = 0.91; ACC_ResNet50_, 0.75 [0.64–0.85] vs. 0.74 [0.62–0.84], *P* = 0.78; AUC_DenseNet121_, 0.79 [0.67–0.88] vs. 0.80 [0.69–0.89], *P* = 0.69; ACC_DenseNet121_, 0.70 [0.57–0.80] vs. 0.74 [0.62–0.84], *P* = 0.43).

The ensemble of the two architectures did not lead to statistically significant improvement on the test set compared to ResNet50, neither for frozen (AUC_Ensemble_ 0.84 [0.73–0.92] vs. AUC_ResNet50_ 0.82 [0.71–0.90], *P* = 0.54; ACC_Ensemble_ 0.75 [0.64–0.85] vs. ACC_ResNet50_ 0.74 [0.62–0.84], *P* = 0.78), nor for unfrozen (AUC_Ensemble_ 0.81 [0.70–0.90] vs. AUC_ResNet50_ 0.82 [0.71–0.91], *P* = 0.70.; ACC_Ensemble_ 0.71 [0.59–0.81], vs. ACC_ResNet50_ 0.75 [0.64–0.85], *P* = 0.40) pre-trained parameters.

**Figure 2:**
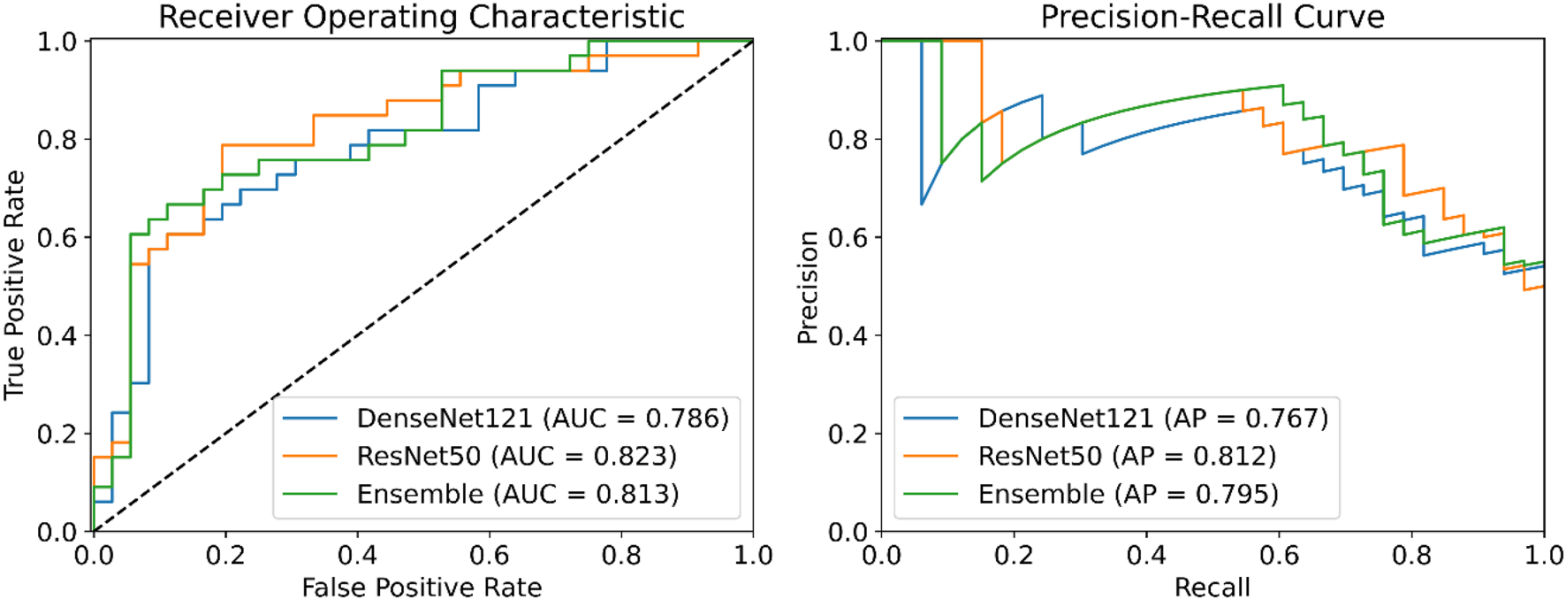
Receiver operating characteristic and precision-recall analysis for the classification performance of DenseNet121 and ResNet50, both trained with unfrozen pre-trained parameters. Abbreviations: *AUC* area under the curve, *AP* average precision.

ROC and precision-recall curves of the models trained with unfrozen pre-trained parameters are given in Fig. 2.

### Highlighted imaging regions according to Grad-CAM

**Figure 3:**
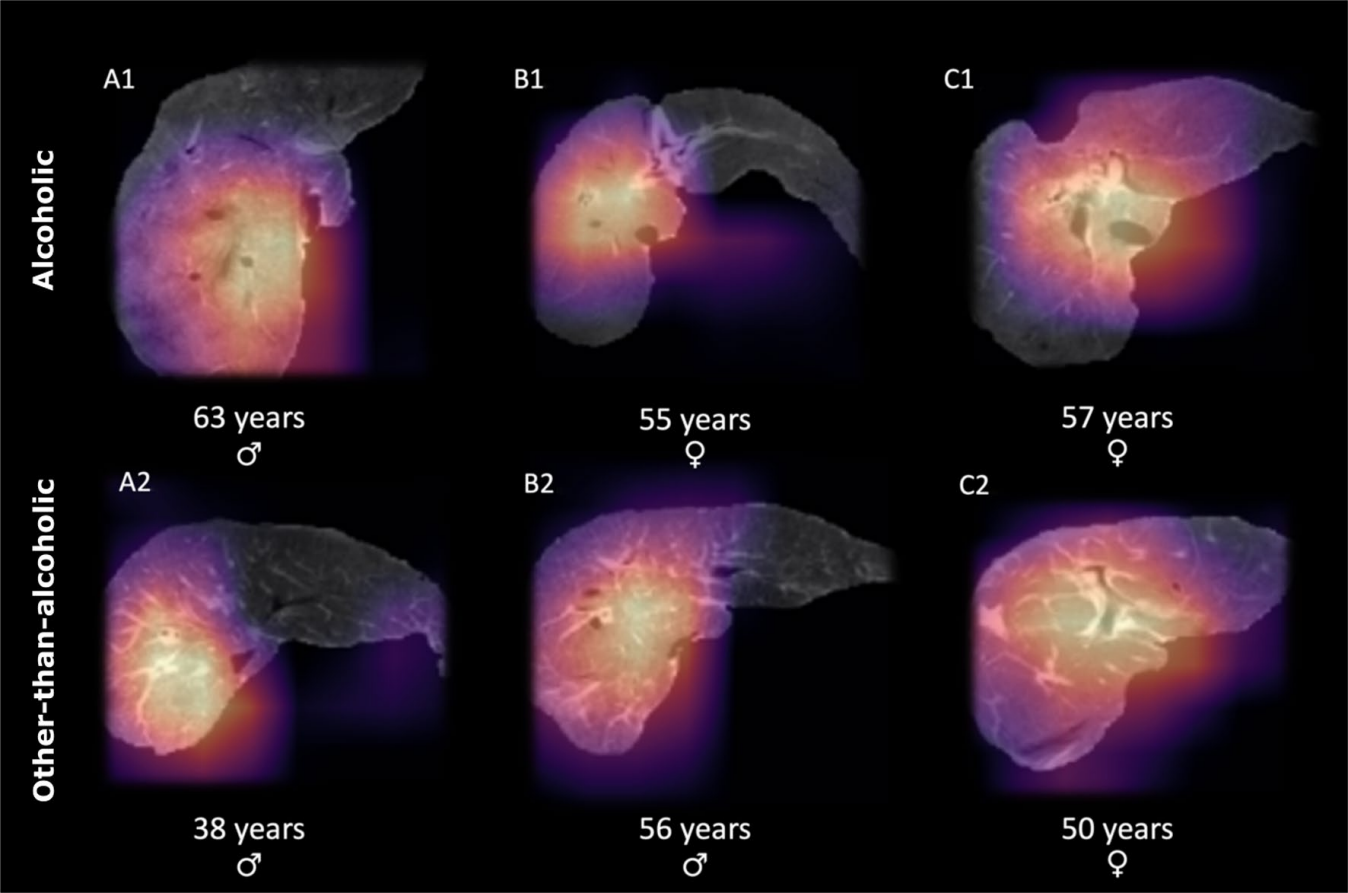
Exemplary images from the study population. ResNet50 trained with unfrozen pre-trained parameters was used for the classification task. Exemplary patients from the test set are provided and imaging regions that were particularly relevant to the classification task are highlighted using the gradient-weighted class activation maps (Grad-CAM) method. Panels A1, B1, C1 provide exemplary patients from the test set with alcoholic liver cirrhosis. In panels A2, B2, C2, images of exemplary patients from the test set with other-than-alcoholic liver cirrhosis are presented. In panels A1, B1, A2, B2, regions within the right liver lobe appeared to be particularly relevant for the classification task, as indicated by Grad-CAM images. In panels C1 and C2, the portal liver region appeared to be most decisive for classification.

The decision process to classify liver cirrhosis as being alcohol related or non-alcohol related was further visualized using Grad-CAM analysis for ResNet50 trained with unfrozen pre-trained parameters. According to Grad-CAM analysis, the right liver lobe (alcoholic liver cirrhosis 42%, 14/33; other-than-alcoholic liver cirrhosis 61%, 22/36) and the portal area (alcoholic liver cirrhosis 30%, 10/33; other-than-alcoholic liver cirrhosis 19%, 7/36) were the imaging regions that were most frequently decisive for the classification process in both groups. Thereby, no significant differences regarding distribution of decisive imaging regions between the two patient groups were observed (Table 3). Exemplary images of the Grad-CAM analysis are provided in Fig. 3.

**Table 3:** Highlighted imaging regions according to gradient-weighted class activation maps (Grad-CAM).

	Alcoholic liver cirrhosis (N = 33)	Other-than-alcoholic liver cirrhosis (N = 36)	*P* value
Right lobe	14 (42%)	22 (61%)	0.12
Left lobe	3 (9%)	3 (8%)	1.00
Portal area	10 (30%)	7 (19%)	0.30
Caudate lobe	4 (12%)	1 (3%)	0.19
background	2 (6%)	3 (8%)	1.00

Results of the visual inspection of Grad-CAM images classified by ResNet50 are provided. Within each segmented image of the test set, highlighted regions were visually rated as being primary located within the right liver lobe, the left liver lobe, the portal area, the caudate lobe, or within image background by one radiologist experienced in abdominal imaging (A.F.).

## Discussion

The purpose of this study was to investigate whether a deep learning-based analysis can aid in differentiating the etiology of liver cirrhosis based on routine clinical T2-weighted MRI. Acceptable to excellent discriminatory ability was found in distinguishing patients with alcoholic and other-than-alcoholic cirrhosis. In a previous study, a ResNet50 with frozen pre-trained ImageNet parameters was proposed for automatic detection of liver cirrhosis on T2-weighted MRI^13^. The results of our proof-of-concept study extend the findings of this previous report and show that deep learning not only enables the detection of cirrhosis, but can also help in identifying the underlying cause of the disease.

Although the ability of the ImageNet pre-trained ResNet50 to discriminate between alcoholic and other-than-alcoholic cirrhosis can be described as excellent^19^, it is inferior to the differentiation of cirrhotic versus non-cirrhotic livers^13^. This may be due to less distinctiveness between imaging criteria indicating different causes of the disease compared to image criteria distinguishing a diseased organ from a non-diseased organ. For instance, it has been described that a hypertrophic appearance of the central hepatic parenchyma/caudate lobe is expected in alcohol-related cirrhosis, but also in primary sclerosing cholangitis and Budd-Chiari syndrome related cirrhosis^11^.

Of both models investigated in the current study, ResNet50 showed higher classification performance on test data. However, the performance was not significantly higher compared to Densenet121. Interestingly, for both CNNs, subsequent training with unfrozen pre-trained parameters did not significantly increase classification performance on test data. This may suggest that the extraction capabilities of general imaging features of the convolutional kernels, learned during the pre-training with the ImageNet database, generalize well to T2-weighted MRI images. An ensemble of the two models trained with unfrozen parameters achieved equal accuracy and a slightly higher AUC compared to ResNet50, however, the difference was not statistically significant. Therefore, no clear advantage was observed by using an ensemble of the two different pre-trained ImageNet architectures.

Grad-CAM-analysis indicate that the imaging morphology of the right liver lobe and caudate lobe seem to comprise particularly relevant information for discrimination of alcoholic from other-than-alcoholic liver cirrhosis. This is in line with previous studies, which describe that the right posterior hepatic notch sign, defined as a sharp liver surface indentation at the posterior boundary of the right and caudate lobe, is considered to be particularly prevalent among patients with alcoholic liver cirrhosis^12,20^. As described above, hypertrophies of the caudate lobe and central hepatic areas are more frequently observed in patients with alcohol-related diseases, but are also seen in other etiologies. To the best of our knowledge, there are currently no studies presenting metrics for the diagnostic accuracy of cirrhosis etiology based on such imaging criteria^11,12^. However, a very recent work investigated a radiomics approach that relates imaging features to the etiology of liver cirrhosis, and also achieved promising results^21^. Unlike the deep learning method presented in the current study, the proposed radiomics approach requires manual definition of region of interests. To date, imaging features have not been used in routine clinical practice to identify alcohol as a cause of cirrhosis.

In clinical routine, liver cirrhosis is typically diagnosed by a combination of characteristic clinical and imaging findings, corresponding laboratory testing and ancillary examinations such as abdominal sonography. Thereby, while this work-up is usually straightforward for virus-related cirrhosis, it may be much more effortful in patients with alcohol-related disease, which in many cases may be diagnosed only by exclusion since there are no specific laboratory findings^22^. Liver biopsy is recommended if cirrhosis etiology is uncertain, but is limited due to its invasive nature, inter-observer variability and potential sampling error^2,23^. Moreover, cirrhosis-related parenchymal changes may hamper or even preclude correct histological analysis^2^.

Compensated liver cirrhosis is frequently asymptomatic; thus, it may be assumed that many patients who undergo routine clinical MRI for other indications may be unaware of a concomitant liver disease. In these patients, a pipeline that automatically identifies tissue alterations and can classify possible disease etiologies has the potential to better guide diagnostic pathways and thus initiate a specific therapy earlier. With the help of deep learning algorithms simple cross-sectional imaging modalities could serve as imaging-based biomarkers for the classification of liver disease in the future. Particularly in alcoholic liver cirrhosis, timely and correct identification of the underlying etiology is crucial, as early abstinence was demonstrated to be the key determinant of long-term outcome^8,24^. Sole clinical assessment of alcoholic liver disease alone might not be trivial in clinical practice because it mostly relies on patients' self-report. In this regard, deep learning applications have the potential to aid diagnosis by extracting also relevant information that may not be readily apparent to the human eye.

Our study has several limitations. The algorithm was developed for binary classification only and does currently not support differentiation of various non-alcohol-related cirrhosis etiologies. Due to the limited number of patients within the respective subclasses and to ensure collectives of comparable size for classification, we decided to pool patients with other-than-alcoholic cirrhosis. Future studies with larger samples of the respective subgroups are needed to substantiate the findings from this proof-of-concept study and to expand its application. The clinical benefit would also be significantly increased by an extension to other etiologies. Especially, NAFLD is becoming the main cause of chronic liver disease in many countries and the detection of metabolic related cirrhosis on cross-sectional imaging should be further explored in future studies. Also, we were not able to analyze possible coexisting etiologies of liver cirrhosis in our explorative analysis, as detailed data on additional risk factors were not available due to the retrospective study design. However, future studies should evaluate the ability of deep learning methods to differentiate overlapping liver disease, such as both alcoholic and non-alcoholic steatohepatitis (BASH). Moreover, we exclusively used single-slice T2-weighted images of segmented livers and ImageNet pre-trained models, as these have been shown to be suitable for the detection of liver cirrhosis in a previous study^13^. Future studies may also address a three-dimensional approach accounting also for extrahepatic manifestations in cirrhotic patients or the use of other imaging sequences for differentiation of etiologies.

In summary, the results of this proof-of-principle study demonstrate that discrimination between alcoholic and other-than-alcoholic cirrhosis based on clinical T2-weighted single-slice images is feasible with acceptable to excellent discrimination ability. This indicates the potential of deep learning for a more comprehensive assessment of diffuse liver disease.

## Supplementary Information

Supplementary Information.

## Data Availability

The data sets analysed in this study are subject to data protection law and are therefore not publicly available.
