# Supplementary material for: Deep learning supports the differentiation of alcoholic and other-than-alcoholic cirrhosis based on MRI
Source: Sci Rep. 2022 May 18;12:8297. doi: 10.1038/s41598-022-12410-2 (PMC9117223; doi:10.1038/s41598-022-12410-2)
Supplement: Supplementary file 1 — Supplementary Information. [file 41598_2022_12410_MOESM1_ESM.docx]

**Supplemental Materials**

S1 Imaging parameters

|  | Training Set (N=396) | | | Test Set (N=69) | | |
| --- | --- | --- | --- | --- | --- | --- |
|  | Mean | Median | Range | Mean | Median | Range |
| Repetition time (ms) | 1986 | 1807 | 1300-4440 | 2062 | 1806 | 1478-3673 |
| Echo time (ms) | 80 | 80 | 80-136 | 80 | 80 | NA |
| Flip angle (°) | 90 | 90 | NA | 90 | 90 | NA |
| Slice thickness (mm) | 4.96 | 5.00 | 4.00-5.00 | 4.96 | 5.00 | 4.00-5.00 |
| Pixel spacing (mm) | 0.82 | 0.88 | 0.49-0.99 | 0.82 | 0.88 | 0.56-1.23 |
| Matrix size | 482 | 432 | 384-768 | 485 | 432 | 320-704 |
| Field of view (mm) | 387 | 380 | 340-490 | 387 | 380 | 360-440 |

Imaging parameters of the used turbo spin echo sequence (T2 MultiVane XD). Abbreviations: NA, not applicable.

S2 Image segmentation and preprocessing

In this study, the segmented livers of single slice images were used. A convolutional neural network (CNN) was applied for liver segmentation with subsequent manual drawing. The CNN followed the principle of the U-net architecture and was developed in a previous work using 713 single slice T2- weighted MRI images at the level of the caudate lobe. 553 images displayed patients with confirmed diagnosis of liver cirrhosis based on clinical or histopathological criteria and 160 displayed patients without history of liver disease. A ResNet34 architecture pre-trained on ImageNet was employed as the encoder. A decoder consisting of twelve convolution layers with rectified linear unit activation and batch normalization replaced the last fully connected layer. After every second convolution layer of the decoder the feature map size is up-scaled using sub-pixel convolution with pixel shuffle arrangement and connected to feature maps of the same size of the ResNet34 encoder [1].

The single slice images were cut to the smallest square image sections containing the segmented liver and all image regions outside the liver mask were written with zero values. Subsequently, the images were interpolated to matrix size 234², which was the median size of the square images containing the livers, thus ensuring minimal image quality loss. Then, the image values in the interval [-$3\sigma3\sigma]$ within the liver mask were transformed to the interval [$0$ $1$]. To provide the same number of channels and the same normalization as the images of the ImageNet dataset, the images were copied into three channels and the mean of the channels were transformed to 0.485, 0.456, 0.406 and the standard deviation of the channels to 0.229, 0.224, 0.225.

All images were augmented by affine transformations during training to improve the robustness of the classification methods against overfitting. Prior to augmentation the 234² sized images were first written centrally into a 256² matrix initialized with zero, in order not to scale and rotate image areas of the liver out of the matrix during augmentation. Then, with a probability of 75%, images were scaled by 0% to 5% and rotating by -10° to 10° along the z-axis while assembling the batches.

S3 Experimental design

ResNet50 and DenseNet121 Convolutional Neural Networks (CNN), both pre-trained on ImageNet, were trained to classify the presence of alcohol-induced liver cirrhosis in images [2,3]. The last fully connected layers of both models were replaced by a fully connected layer with two neurons, matching the number of classification classes. Both models were trained with two methods.

In the first method, the models served as feature extractor, i.e. only the fully connected layer was trained and all parameters of the pre-trained convolutional layers were kept frozen. In the second method, the parameters of the pre-trained convolutional layers were subsequently unfrozen as described below. Early stopping was applied for implicit regularization by applying receiver operating characteristic analysis to the validation set during training and storing the model state with the highest area under the curve (AUC) [4]. In both training methods (frozen/unfrozen), the learning rate (LR) and momentum followed a cyclical change during each training [5]. Training was performed with the Adam optimization algorithm and the cross-entropy loss function [6]. A weight decay of 15 was implemented as described by Loshchilov et al. [7].

In the training with frozen pre-trained parameters (training the last fully connected layer only), the LR was increased to a maximum value of ${5*10}^{-3}$ until epoch 72 was reached, starting from the maximum value divided by 25. Then the LR was dropped towards zero by cosine annealing until epoch 240. The momentum was dropped from 0.95 to 0.85 and then raised back to 0.95.

The subsequent training with unfrozen pre-trained parameters consisted of three stages that started from the model state with the highest validation AUC of its previous stages. All stages followed the same scheme of a cyclic LR and momentum, but where the learning rate (LR) was modified, as detailed below.

In the first stage, the pre-trained parameters of the convolutional layers were unfrozen. In addition to the previously described frozen training, 240 epochs were trained, however the maximum LR of the individual CNN layers were varied linearly from ${10}^{-6}$ to ${10}^{-3}$ (from the first to the output layer). In the second stage, additional 240 epochs were trained with a maximum LR of the individual CNN layers varying linearly from ${10}^{-5}$ to ${10}^{-3}$. In the third stage, 240 epochs $were trained$ with a maximum LR of ${10}^{-4}$for all layers of the CNN. In all stages early stopping by validation AUC was used, to try to improve the model state of the previous stage.

All experiments were performed in pytorch and the application programming interface fastai [8,9]. The training was performed on a Nvidia Titan RTX Graphics Processing Unit (GPU) with 24 gigabyte video memory, using mixed precision training. For all experiments, the batch size was 158.

**References**

1. Shi W. *et al.* Real-Time Single Image and Video Super-Resolution Using an Efficient Sub-Pixel Convolutional Neural Network. In: *CVPR 2016,* 1874-1883 (2016).

2. He K., Zhang X., Ren S. & Sun J. Deep Residual Learning for Image Recognition. In: *CVPR 2016*, 770-778 (2016).

3. Huang G., Liu Z., van der Maaten L. & Weinberger K. Q. Densely Connected Convolutional Networks. In: *CVPR 2017*, 4700-4708 (2017).

4. Zhang C., Bengio S., Hardt M., Recht B. & Vinyals O. Understanding deep learning (still) requires rethinking generalization. *Commun. ACM* **64**(3), 107-115 (2021).

5. Smith L. N. A disciplined approach to neural network hyper-parameters: Part 1 -- learning rate, batch size, momentum, and weight decay. Preprint at https://arxiv.org/abs/1803.09820 (2018).

6. Kingma D. P. & Ba J. Adam: A Method for Stochastic Optimization. Preprint at https://arxiv.org/abs/1412.6980 (2014).

7. Loshchilov I. & Hutter F. Decoupled Weight Decay Regularization. Preprint at https://arxiv.org/abs/1711.05101 (2017).

8. Paszke A. *et al.* PyTorch: An Imperative Style, High-Performance Deep Learning Library. In: *Adv. Neural Inf. Process. Syst.* 32*,* 8024-8035 (2019).

9.  Howard J. & Gugger S. Fastai: a layered API for deep learning. *Information* ***11***(2), 108 (2020).
